# Final approval for corticosteroids in severe CAP? For sure, in septic shock

**DOI:** 10.1186/s13054-023-04613-4

**Published:** 2023-09-04

**Authors:** Ignacio Martin-Loeches, Blin Nagavci, Antoni Torres

**Affiliations:** 1 Department of Intensive Care Medicine, Multidisciplinary Intensive Care Research Organization (MICRO), Leinster, Dublin, D08NYH1 Ireland; 2 Independent Researcher - Methodologist, I. Boletini 850000, Gjakova, Kosovo; 3 Pulmonary Intensive Care Unit, Respiratory Institute, Hospital Clinic of Barcelona, IDIBAPS (Institut d’Investigacions Biomèdiques August Pi I Sunyer), University of Barcelona, ICREA CIBERes, 08380 Barcelona, Spain

The use of corticosteroids in the care of critically ill patients has been the subject of continuous research efforts with discrepant results. In sepsis, several “swings of the pendulum” for administering such drugs have occurred in the last two decades. [1]. A continuous and growing interest is in determining their role in a common source of sepsis: community-acquired pneumonia (CAP) patients. Some studies in the last decade aimed to determine if corticosteroids resulted in a clinical benefit to the patients with the most severe clinical presentation and highest mortality of CAP: Severe community-acquired pneumonia (sCAP). Corticosteroids have already been shown to be beneficial in those with high systemic inflammatory response [2] and acute respiratory distress syndrome (ARDS) [3]. Corticosteroids may also be useful in patients with underlying chronic obstructive pulmonary disease (COPD) or asthma, who may have a more severe inflammatory response to sCAP. However, other studies have not found any significant benefit of corticosteroid therapy in CAP in mortality or treatment failure [4] and have raised concerns about potential harm, including increased risk of secondary infections and delayed resolution for pneumonia [5].

A recent systematic review and meta-analysis conducted by Jheng‑Yan Wu et al., in *Critical Care,* assessed the efficacy and safety of adjunctive corticosteroids in sCAP [6]. The authors included the same studies analysed in the ERS/ESICM/ESCMID/ALAT guidelines for managing severe community-acquired pneumonia [7], with the addition of the recent trial by Dequin et al. [8] Jheng‑Yan Wu et al. reported that corticosteroids in patients with sCAP could provide survival benefits and improve clinical outcomes (mortality, duration of mechanical ventilation). Furthermore, they suggest that corticosteroids may have a role in treating sCAP even without initial septic shock. As authors of the guidelines on sCAP [7], we have updated the meta-analyses of the sCAP guidelines, including the original trials [2, 9–13], adding the recent randomised controlled trial by Dequin et al. [8] (Figs. 1, 2, 3, 4, 5, 6, 7). While the meta-analyses of Jheng-Yan Wu et al. aligned closely with our results in technical terms, the interpretation did not. We consider that their interpretation is not entirely accurate for two main reasons:

1. Inconclusiveness of evidence: The trial recently published by Dequin et al. [8], which randomised 800 patients to either corticosteroids or placebo, reported a statistically significant reduction in deaths (5.6%) in the corticosteroids group. However, this trial had some particularities. The corticosteroid administered was hydrocortisone, and patients with septic shock at baseline were not included in the trial. They also found a reduced risk of intubation or receiving vasopressors in the hydrocortisone group. This was considered indirect data for shock in our meta-analyses. Therefore, data for Dequin et al. was added only in shock (total), as it is unclear if these patients had septic or other types of shock (Fig. 2). This trial presents contradictory findings compared to the second-largest trial published by Meduri et al., which reported no benefits for corticosteroids [13]. This randomised study with 600 patients used a different corticosteroid, methylprednisolone—adding that there have previously been mixed results for corticosteroid drugs in cases of sCAP. The disparities in their outcomes could be due to differing dosage procedures, antibiotic levels, and changes in how sCAP is identified and classified according to organ failure. While the two largest trials contradict each other, the remaining ones are relatively small and inconclusive. This shows that the evidence is far from conclusive on this topic, a conclusion also reached by Jheng‑Yan Wu et al.
2. The risk of bias (RoB) in the included trials: In the meta-analysis performed by Jheng‑Yan Wu et al., we consider that authors were less strict in their RoB assessments by assessing all trials to have a low risk of bias in most domains. We strongly believe that this is not the case. In the sCAP guidelines (and our updated) meta-analyses, RoB was assessed more stringently. For instance, in the ICU mortality outcome, four out of seven trials were rated to have unclear RoB in allocation concealment and blinding of outcomes (Fig. 1). The unclear RoB in most trials should cause concern and fuel some reluctance when suggesting corticosteroids for all sCAP patients. In addition, we included some subgroup analyses that Jheng-Yan Wu et al. did not evaluate, such as the development of shock, mechanical ventilation-free days and cardiac complications.

**Fig. 1:**
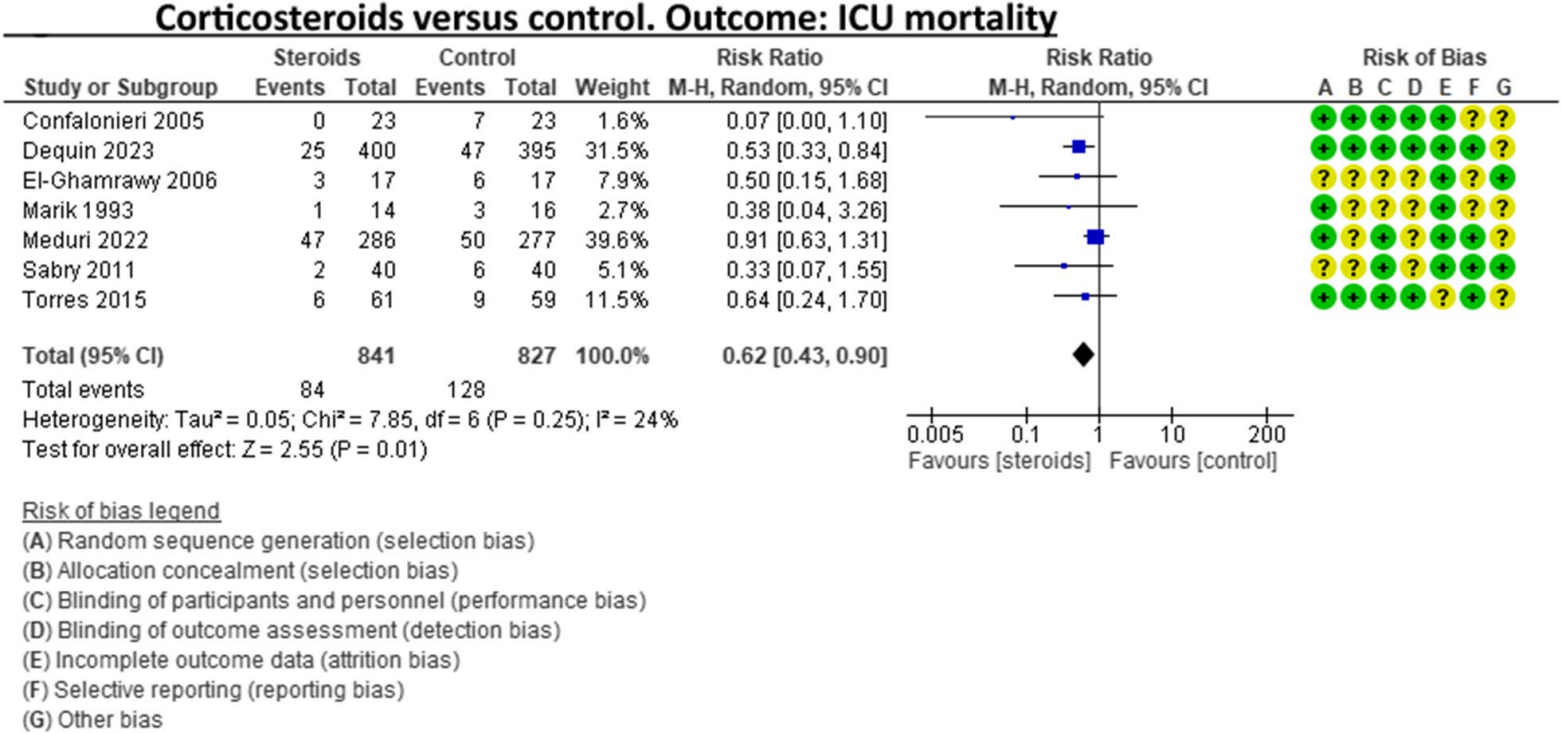
Corticosteroids versus control. Outcome: ICU mortality

**Fig. 2:**
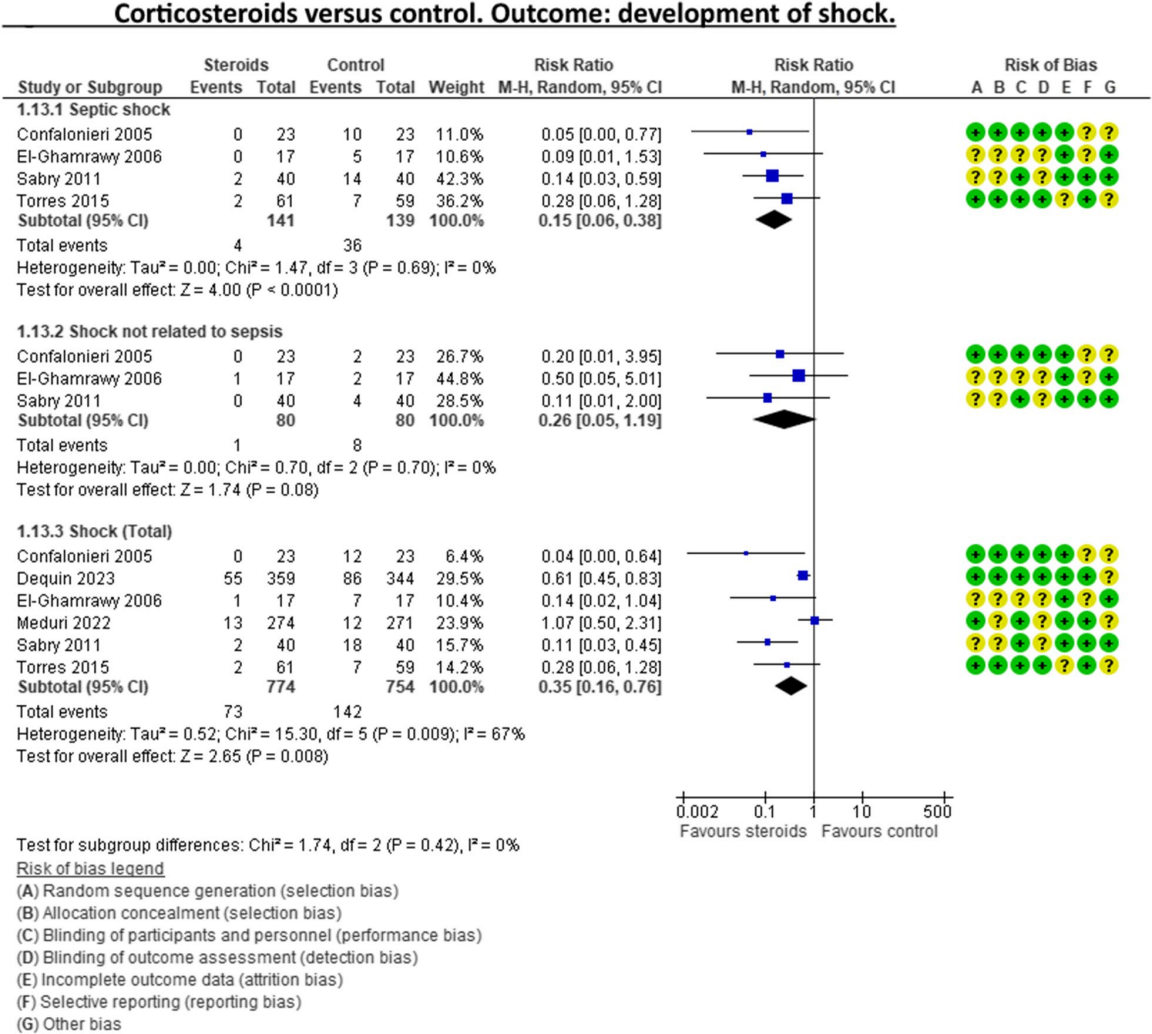
Corticosteroids versus control. Outcome: development of shock

**Fig. 3:**
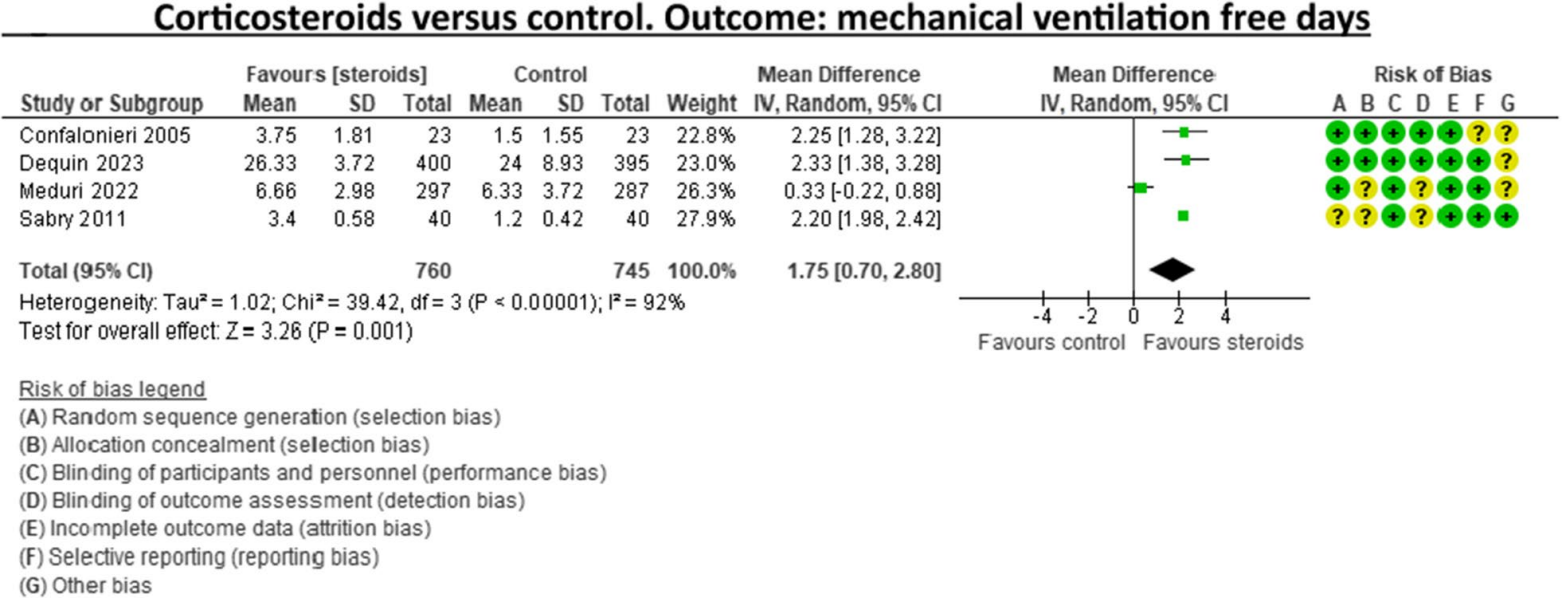
Corticosteroids versus control. Outcome: mechanical ventilation-free days

**Fig. 4:**
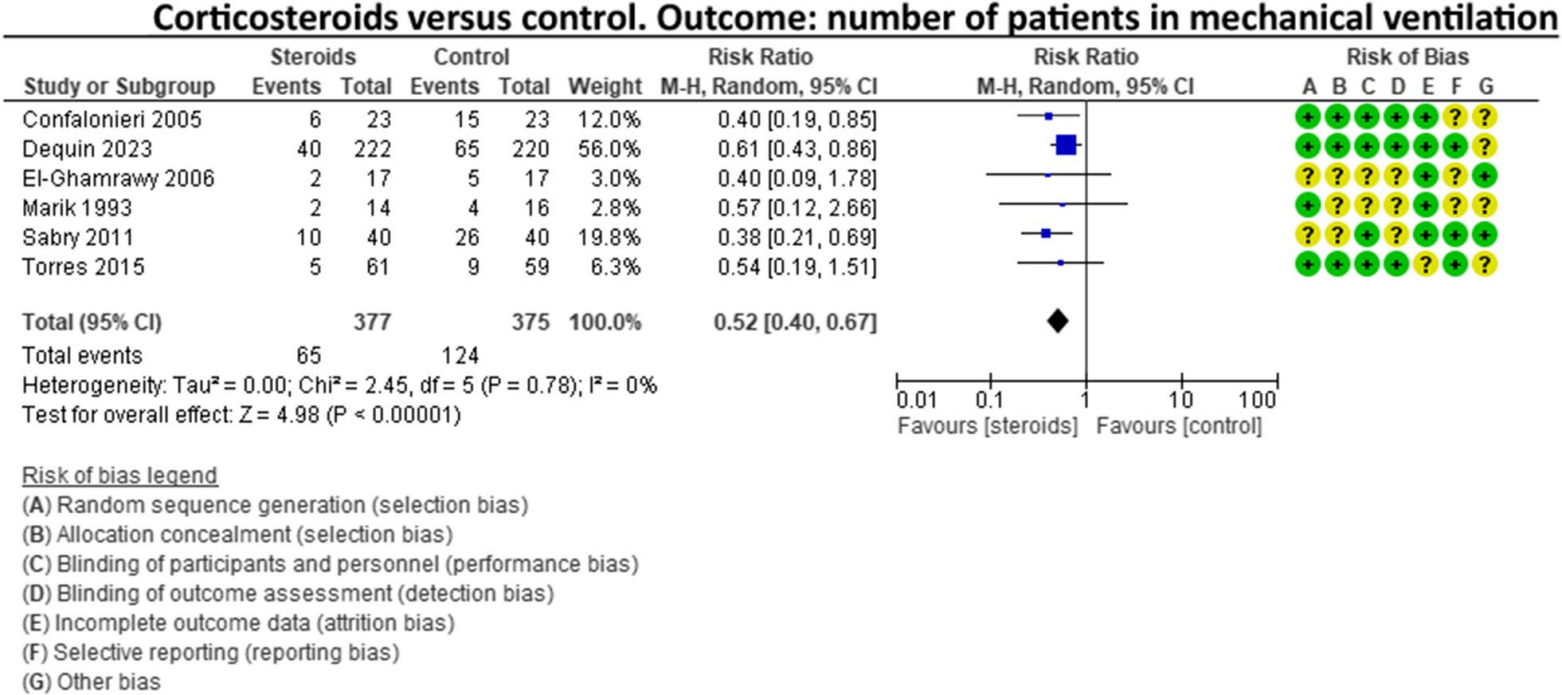
Corticosteroids versus control. Outcome: number of patients in mechanical ventilation

**Fig. 5:**
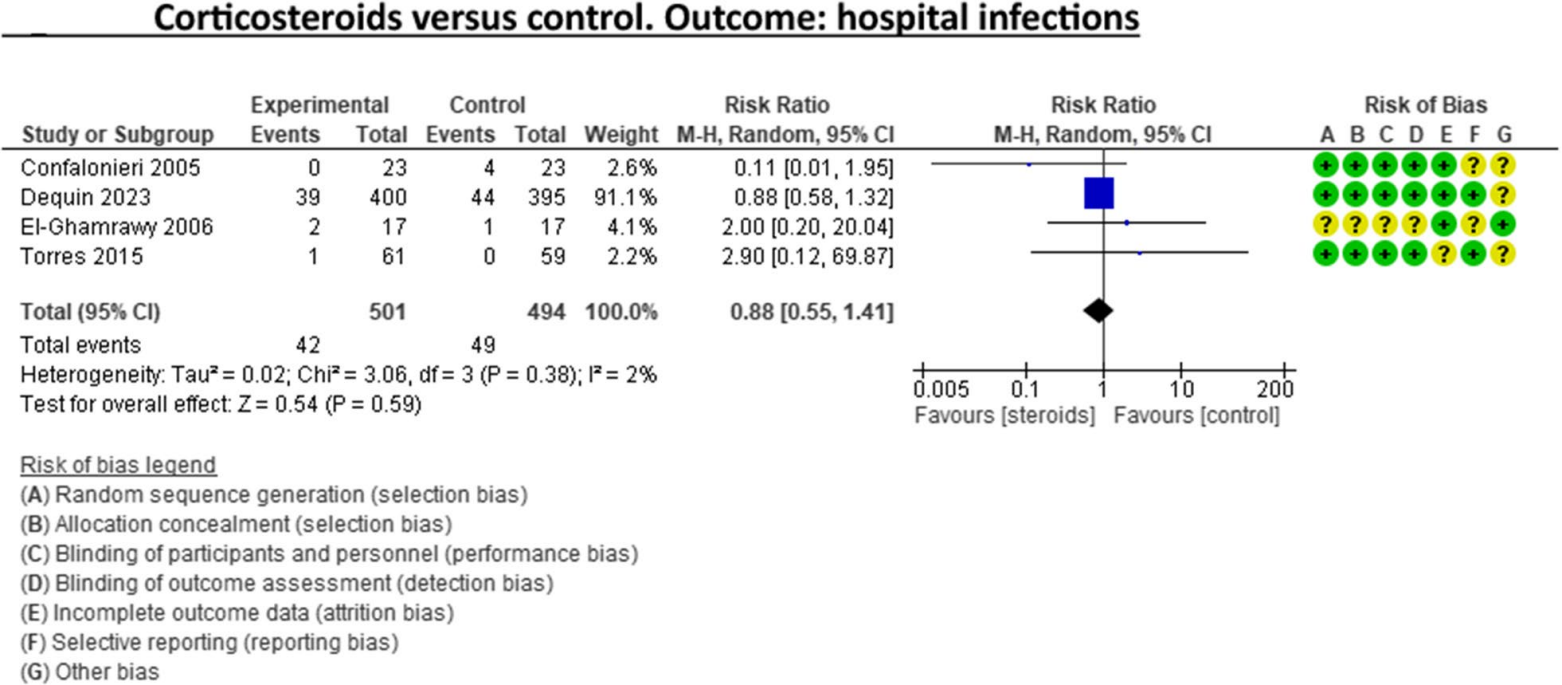
Corticosteroids versus control. Outcome: hospital infections

**Fig. 6:**
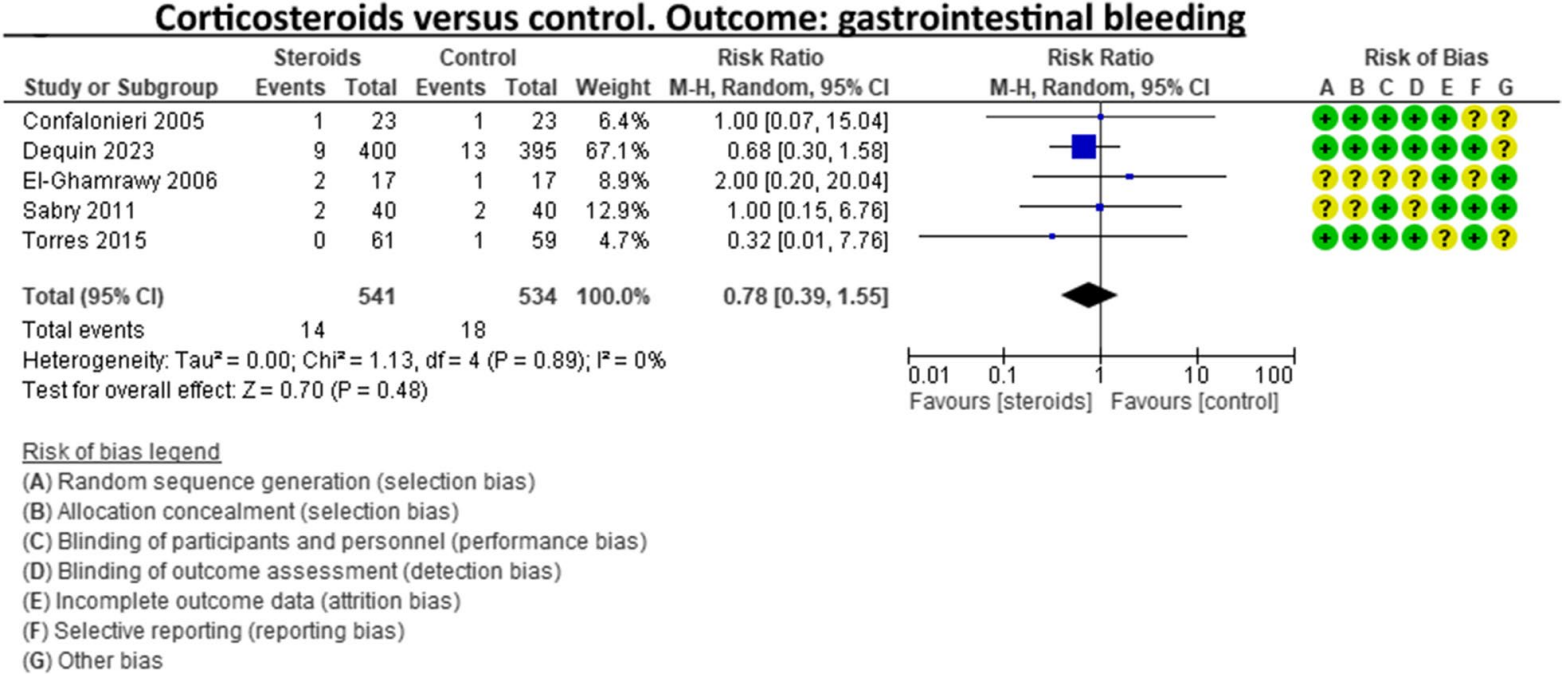
Corticosteroids versus control. Outcome: gastrointestinal bleeding

**Fig. 7:**
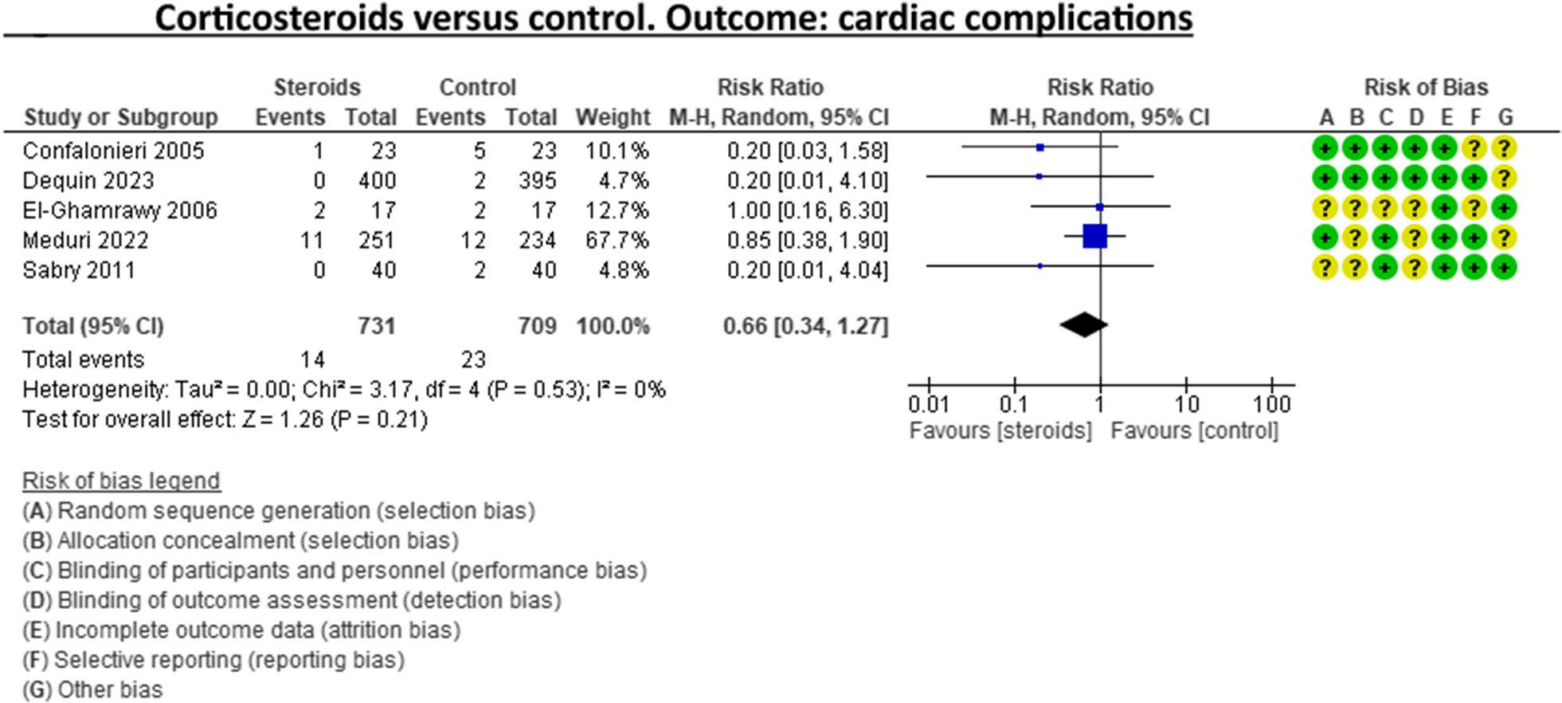
Corticosteroids versus control. Outcome: cardiac complications

Although meta-analyses show some positive effects, careful consideration should be given to the limitations of each included study. Meta-analyses remain valuable tools, but they require careful interpretations. Otherwise, a non-expert in the field or even artificial intelligence software/s could conduct them in the future. We agree there is a signal for better outcomes when corticosteroids are used in sCAP, but the evidence is not yet definitive. Further research is required to establish a more conclusive understanding of the effectiveness and appropriate use of corticosteroids in the treatment of sCAP, especially in patient subgroups, e.g. in elderly patients where corticosteroids could be linked to deleterious effects such as opportunistic infections, bleeding, and neuropsychiatric side effects. In other words, the decision to use or not use corticosteroids in treating sCAP should be based on individual patient factors, including the severity of illness, underlying medical conditions, and potential risks and benefits of therapy.

We acknowledge the overall mortality benefit in patients receiving corticosteroids for sCAP. However, the clearest signal for using corticosteroids is in sCAP patients with shock, as it has been recently recommended by the recent ERS/ESICM/ESCMID/ALAT sCAP guidelines.

## Data Availability

Not applicable.

## References

[1] Vincent JL (2008). Steroids in sepsis: another swing of the pendulum in our clinical trials. Crit Care.

[2] Torres A, Sibila O, Ferrer M, Polverino E, Menendez R, Mensa J, Gabarrus A, Sellares J, Restrepo MI, Anzueto A (2015). Effect of corticosteroids on treatment failure among hospitalized patients with severe community-acquired pneumonia and high inflammatory response: a randomized clinical trial. JAMA.

[3] Villar J, Ferrando C, Martínez D, Ambrós A, Muñoz T, Soler JA, Aguilar G, Alba F, González-Higueras E, Conesa LA (2020). Dexamethasone treatment for the acute respiratory distress syndrome: a multicentre, randomised controlled trial. Lancet Respir Med.

[4] Saleem N, Kulkarni A, Snow TAC, Ambler G, Singer M, Arulkumaran N (2023). Effect of corticosteroids on mortality and clinical cure in community-acquired pneumonia: a systematic review, meta-analysis, and meta-regression of randomized control trials. Chest.

[5] Reyes LF, Rodriguez A, Bastidas A, Parra-Tanoux D, Fuentes YV, García-Gallo E, Moreno G, Ospina-Tascon G, Hernandez G, Silva E (2022). Dexamethasone as risk-factor for ICU-acquired respiratory tract infections in severe COVID-19. J Crit Care.

[6] Wu J-Y, Tsai Y-W, Hsu W-H, Liu T-H, Huang P-Y, Chuang M-H, Liu M-Y, Lai C-C (2023). Efficacy and safety of adjunctive corticosteroids in the treatment of severe community-acquired pneumonia: a systematic review and meta-analysis of randomized controlled trials. Crit Care.

[7] Martin-Loeches I, Torres A, Nagavci B, Aliberti S, Antonelli M, Bassetti M, Bos LD, Chalmers JD, Derde L, de Waele J (2023). ERS/ESICM/ESCMID/ALAT guidelines for the management of severe community-acquired pneumonia. Intensive Care Med.

[8] Dequin PF, Meziani F, Quenot JP, Kamel T, Ricard JD, Badie J, Reignier J, Heming N, Plantefève G, Souweine B (2023). Hydrocortisone in severe community-acquired pneumonia. N Engl J Med.

[9] Sabry NA (2011). Omar EE-D: corticosteroids and ICU course of community acquired pneumonia in Egyptian settings. Pharmacol Pharm.

[10] El-Ghamrawy A, Shokeir M, Esmat A (2006). Effects of low-dose hydrocortisone in ICU patients with severe community-acquired pneumonia. Egypt J Chest.

[11] Confalonieri M, Urbino R, Potena A, Piattella M, Parigi P, Puccio G, Della Porta R, Giorgio C, Blasi F, Umberger R (2005). Hydrocortisone infusion for severe community-acquired pneumonia: a preliminary randomized study. Am J Respir Crit Care Med.

[12] Marik P, Kraus P, Sribante J, Havlik I, Lipman J, Johnson DW (1993). Hydrocortisone and tumor necrosis factor in severe community-acquired pneumonia: a randomized controlled study. Chest.

[13] Meduri GU, Shih MC, Bridges L, Martin TJ, El-Solh A, Seam N, Davis-Karim A, Umberger R, Anzueto A, Sriram P (2022). Low-dose methylprednisolone treatment in critically ill patients with severe community-acquired pneumonia. Intensive Care Med.

